# Supplementary figures and images for: Quantitative mapping of proteasome interactomes and substrates using ProteasomeID
Source: eLife. 2024 Sep 4;13:RP93256. doi: 10.7554/eLife.93256 (PMC11374303; doi:10.7554/eLife.93256)

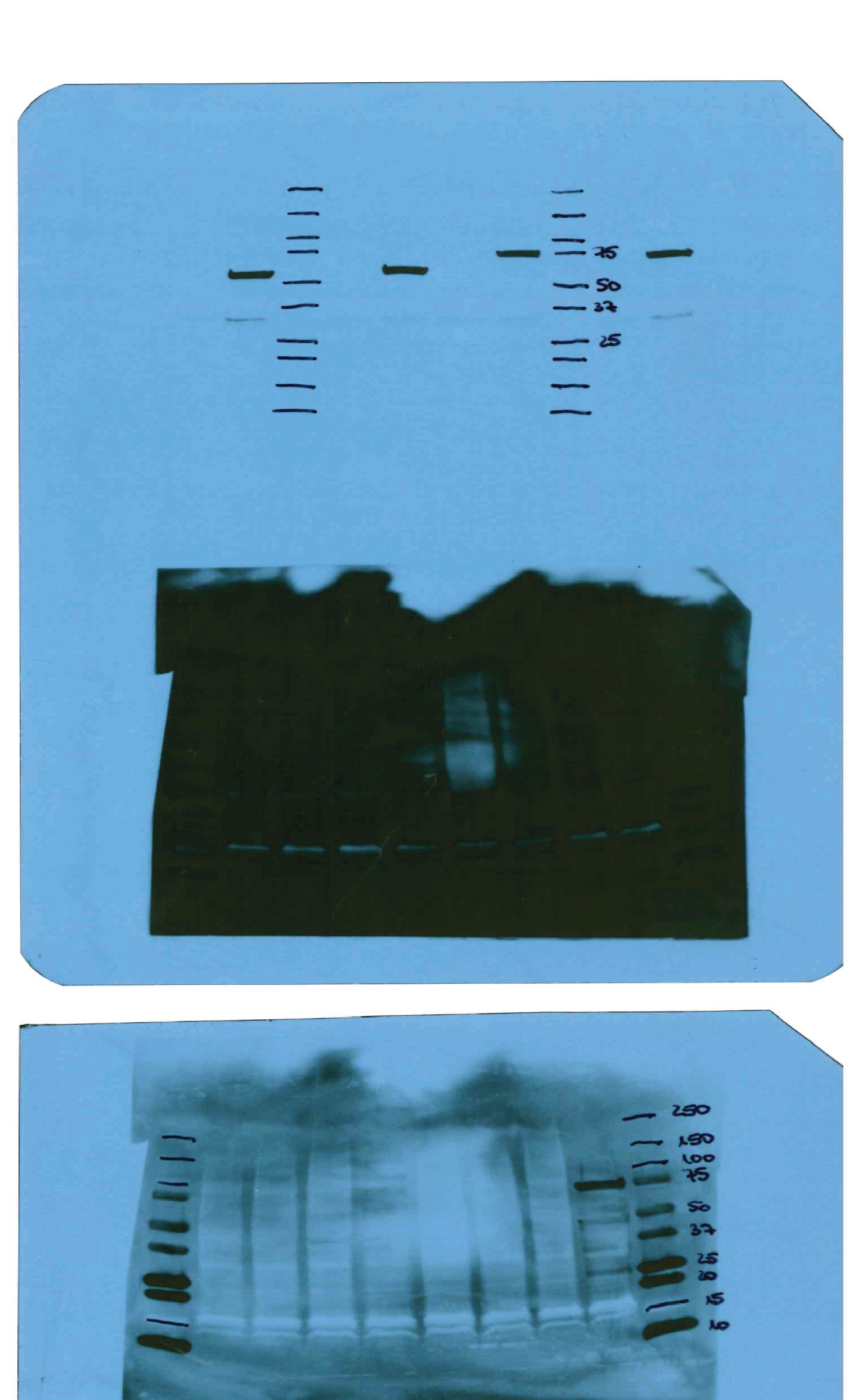

Supplement: Figure 1—source data 1. [file elife-93256-fig1-data1.zip › Figure1_SourceData_1/160615_Fig1b_PSMA4_Flag.jpg]

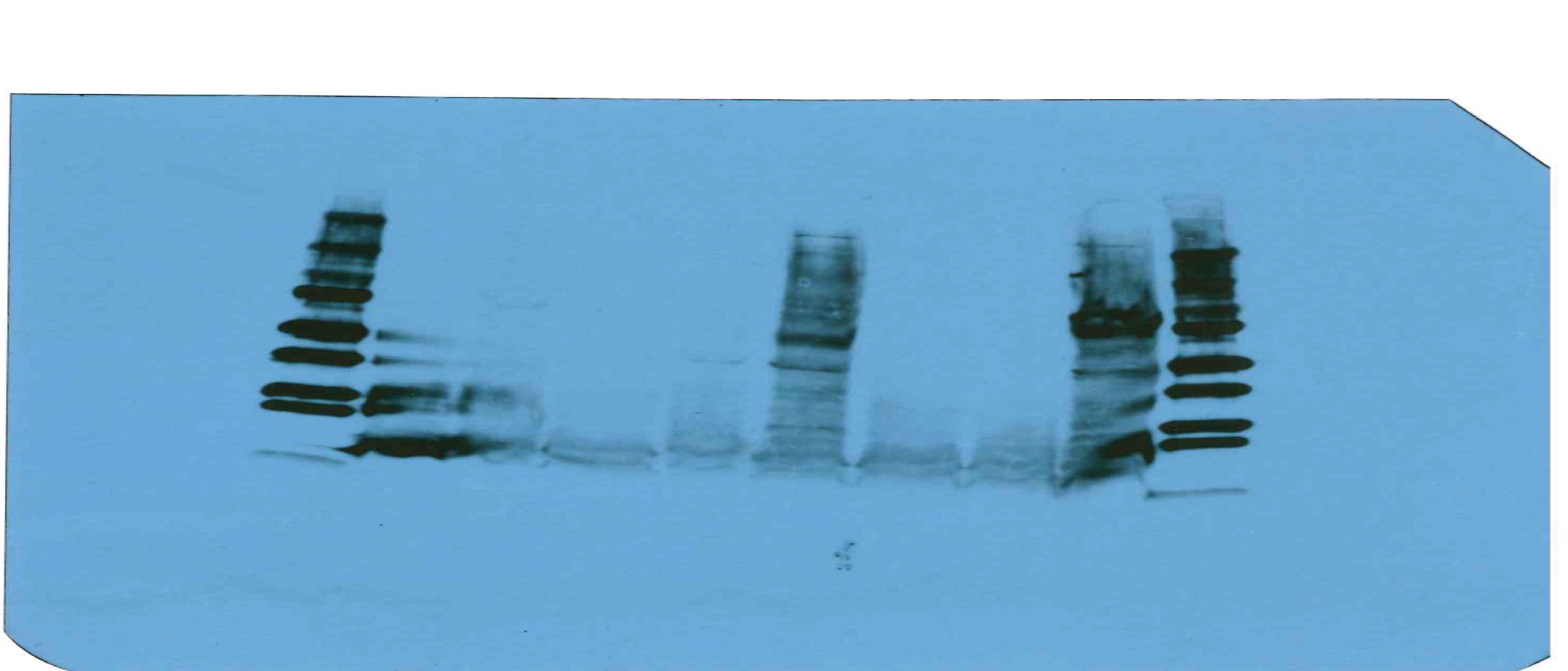

Supplement: Figure 1—source data 1. [file elife-93256-fig1-data1.zip › Figure1_SourceData_1/160718_Fig1b_PSMA4_streptavidin.jpg]

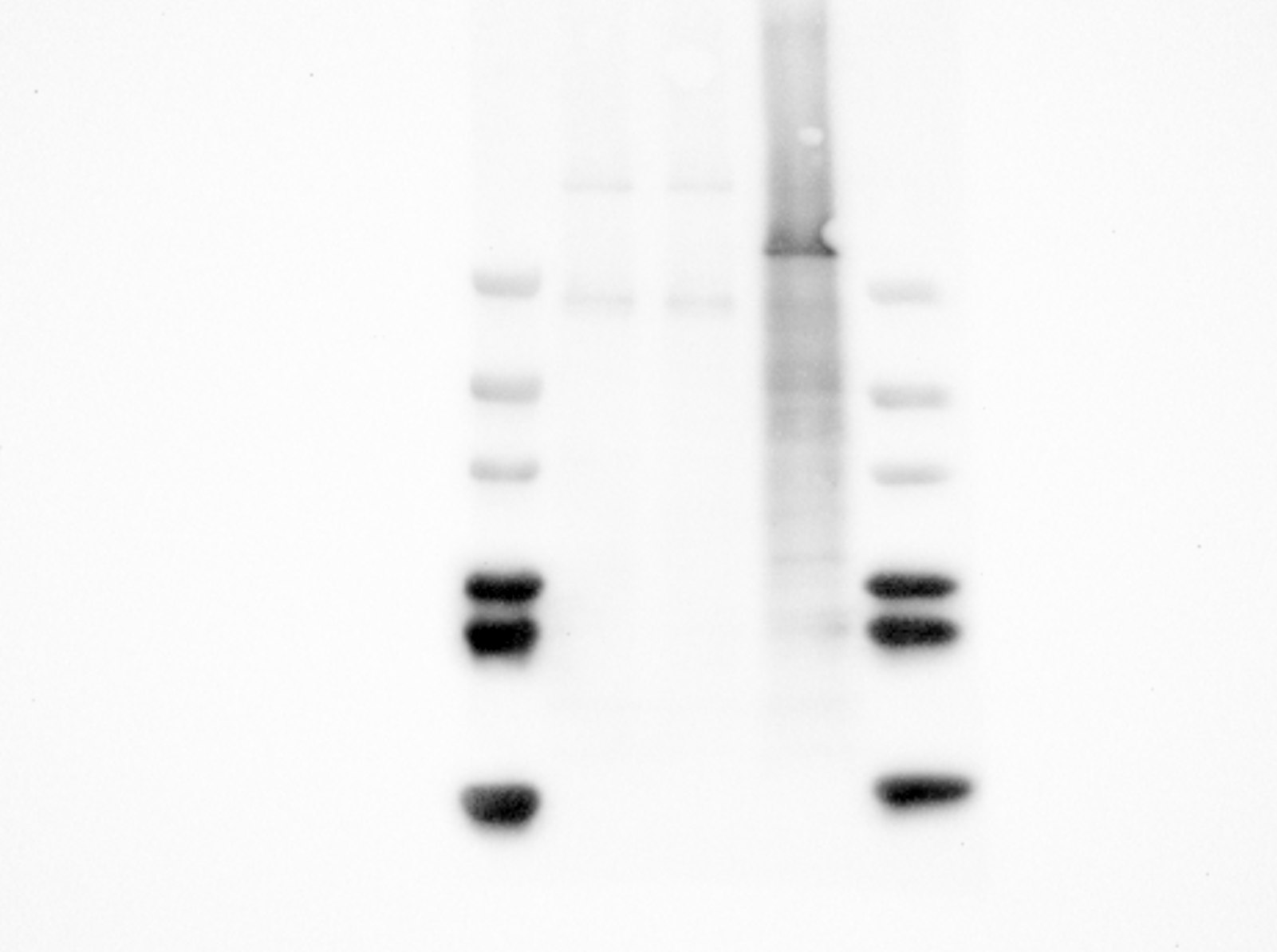

Supplement: Figure 1—source data 1. [file elife-93256-fig1-data1.zip › Figure1_SourceData_1/230428_Fig1b_PSMD3_Streptavidin_auto.tif]

Figure 2a

ponceau S

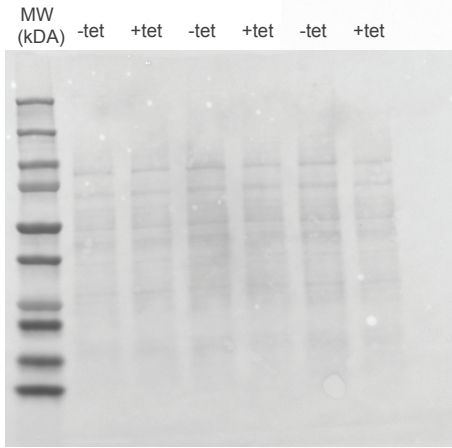

anti-PSMA4

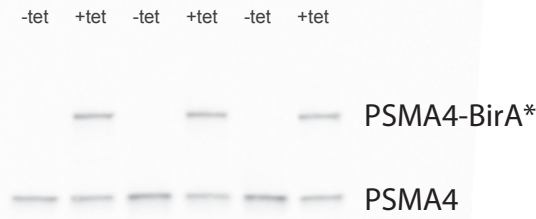

Supplement: Figure 2—source data 2. [file elife-93256-fig2-data2.zip › Figure2_SourceData_2/Figure_2a.pdf]

Figure 2f

ponceau S

anti-c-myc

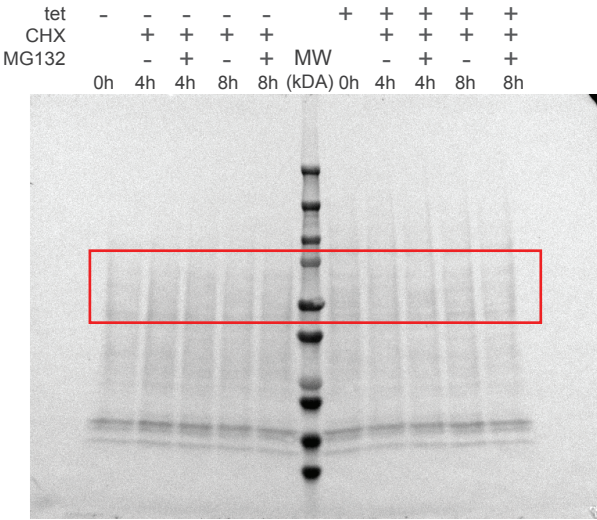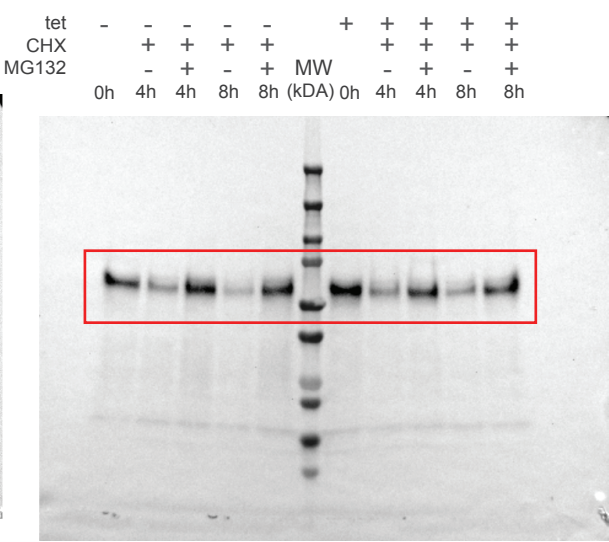

Supplement: Figure 2—source data 2. [file elife-93256-fig2-data2.zip › Figure2_SourceData_2/Figure_2f.pdf]

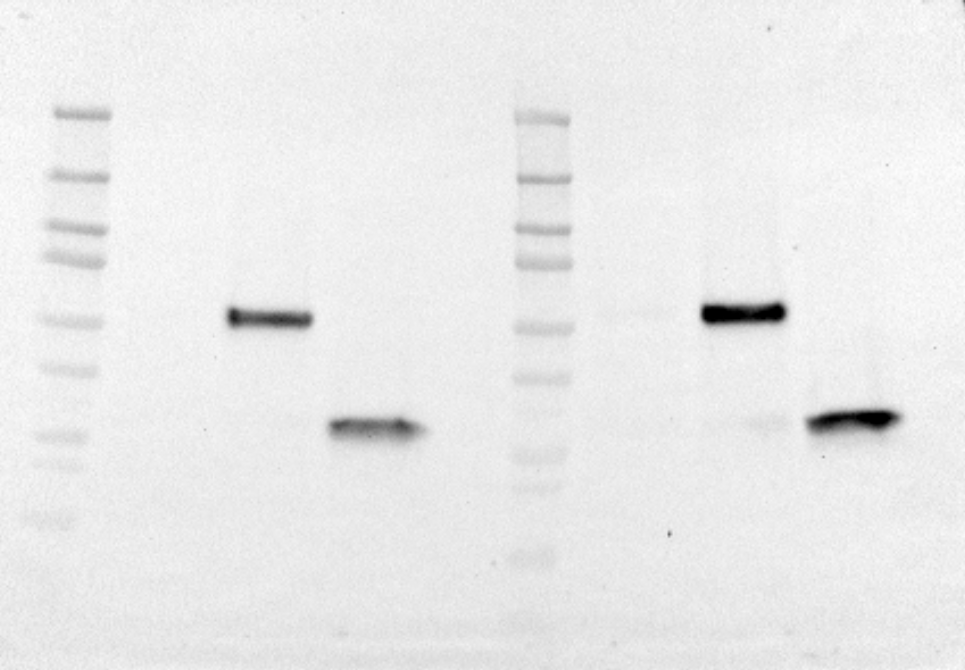

Supplement: Figure 2—figure supplement 1—source data 1. [file elife-93256-fig2-figsupp1-data1.zip › Figure2_figure_supplement1_SourceData_1/BirA/Common User account Ori 2024-02-01_09h26m25s_Exposure_152.3sec+Common User account Ori 2024-02-01_09h34m18s_ladder.tif]

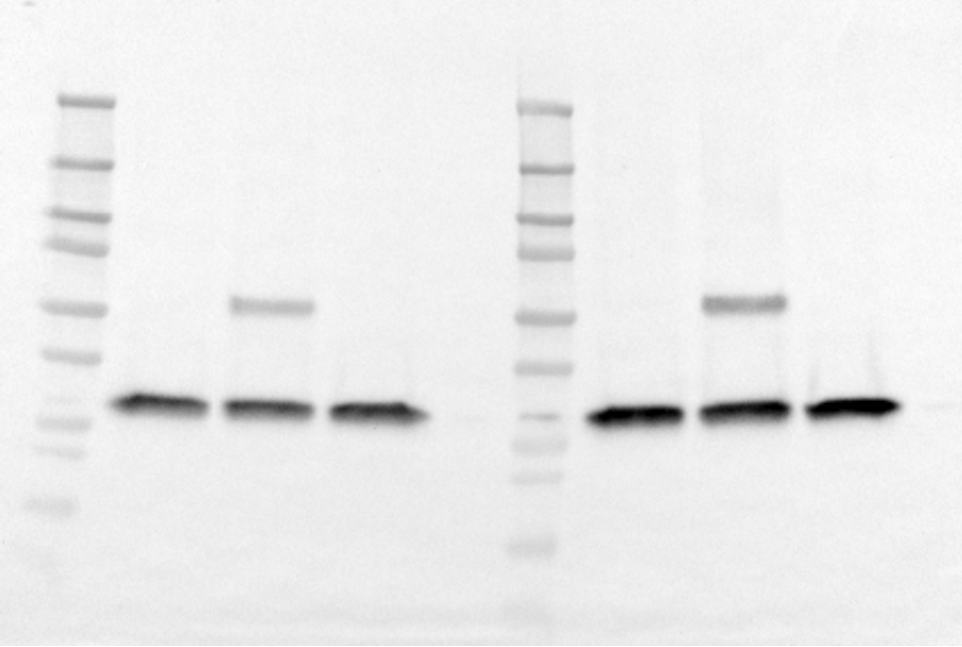

Supplement: Figure 2—figure supplement 1—source data 1. [file elife-93256-fig2-figsupp1-data1.zip › Figure2_figure_supplement1_SourceData_1/PSMA4/Common User account Ori 2024-01-31_08h41m59s_Exposure_42.3sec+Common User account Ori 2024-01-31_08h48m43s_ladder.tif]

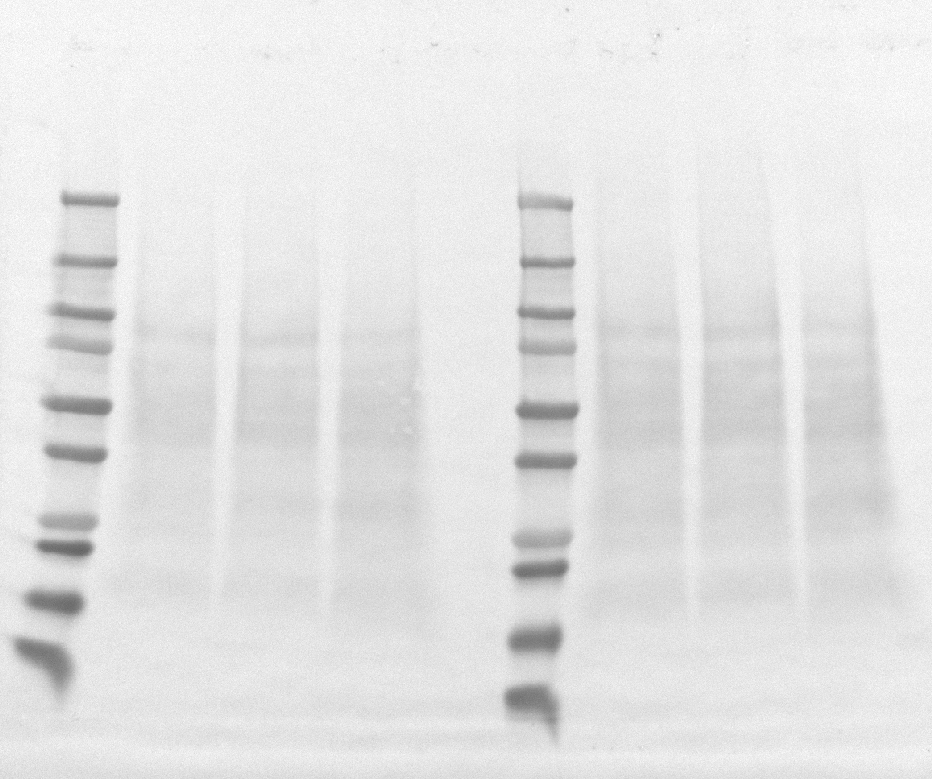

Supplement: Figure 2—figure supplement 1—source data 1. [file elife-93256-fig2-figsupp1-data1.zip › Figure2_figure_supplement1_SourceData_1/Ponceau/Common User account Ori 2024-01-29_09h35m26s_ponceau.tif]

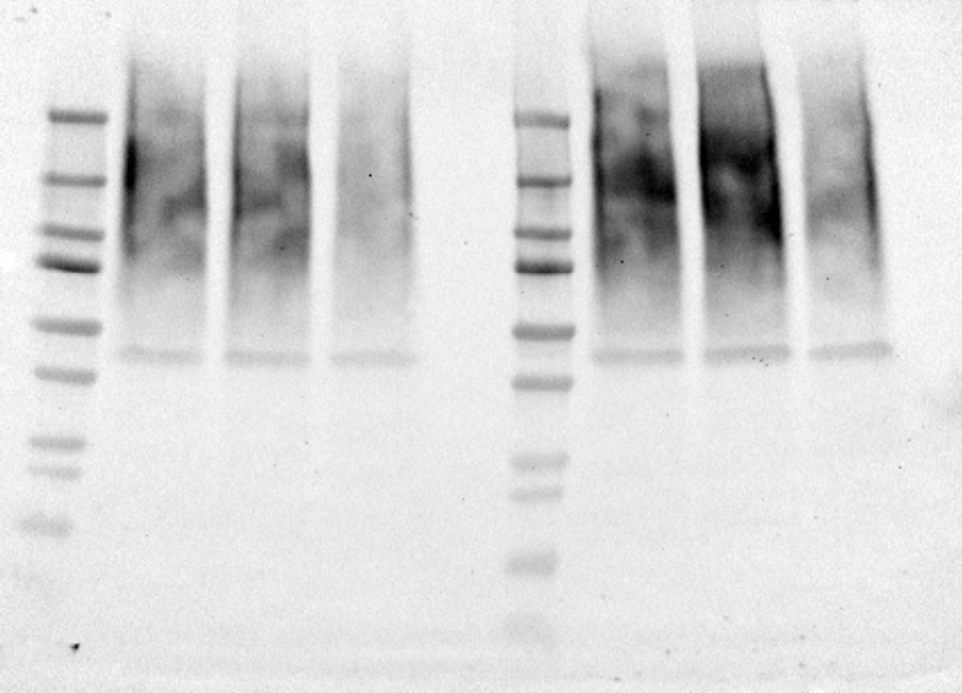

Supplement: Figure 2—figure supplement 1—source data 1. [file elife-93256-fig2-figsupp1-data1.zip › Figure2_figure_supplement1_SourceData_1/K48 Ubi/Common User account Ori 2024-01-30_10h17m50s_Exposure_400.0sec+Common User account Ori 2024-01-30_10h25m44s_ladder.tif]

Figure 2 figure supplement 1d

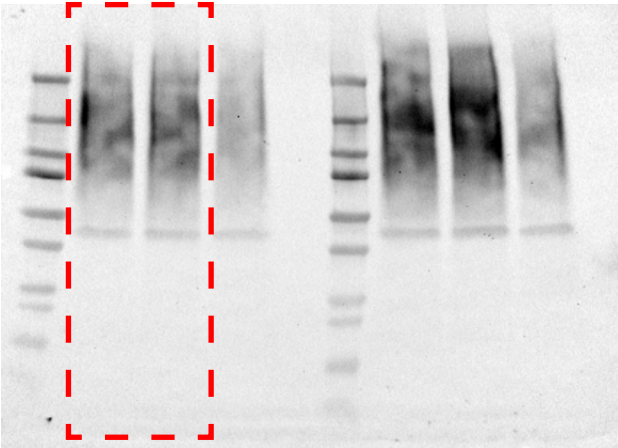

K48-Ubi

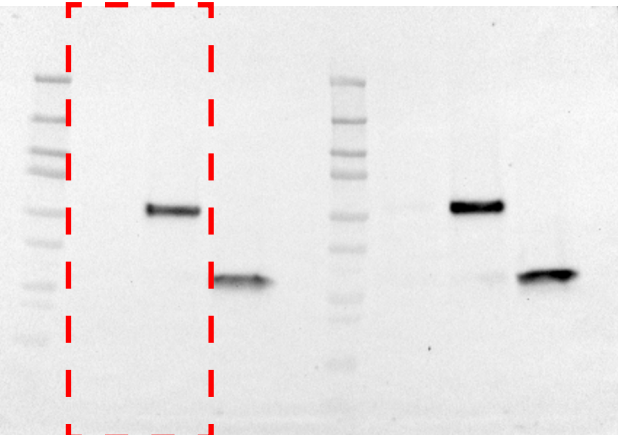

anti-BirA

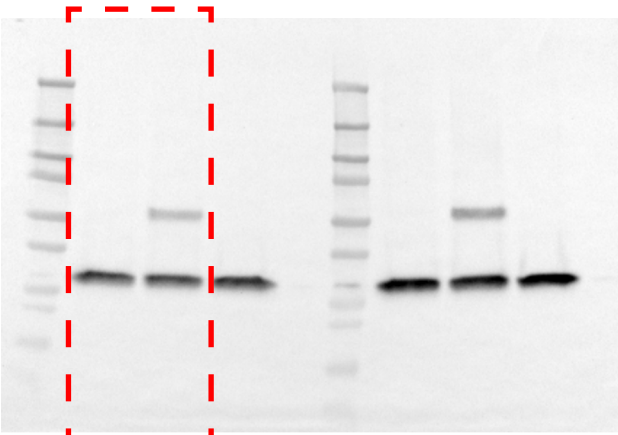

anti-PSMA4

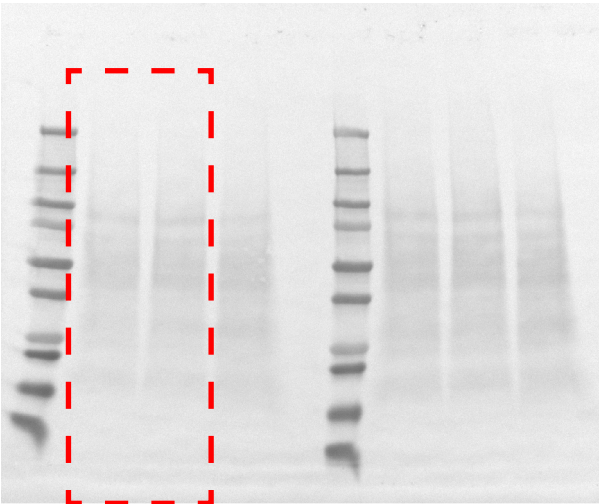

PonceauS

Supplement: Figure 2—figure supplement 1—source data 2. [file elife-93256-fig2-figsupp1-data2.zip › Figure2_figure_supplement1_SourceData_2/Figure2_figure_supplement1d_uncropped_gels.pdf]

Figure 4 figure supplement 1f

ponceau S

anti-Ubi-K48

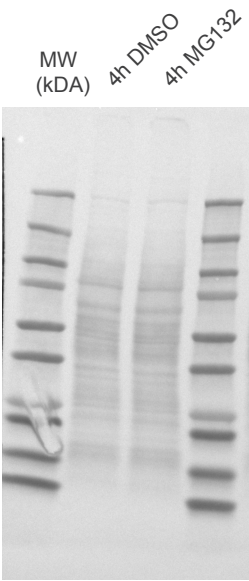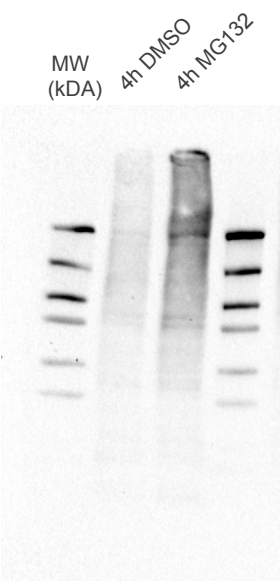

Supplement: Figure 4—figure supplement 1—source data 2. [file elife-93256-fig4-figsupp1-data2.zip › Figure4_SourceData_2/Figure4_S1f.pdf]

Figure 4 figure supplement 1a

anti-GAPDH

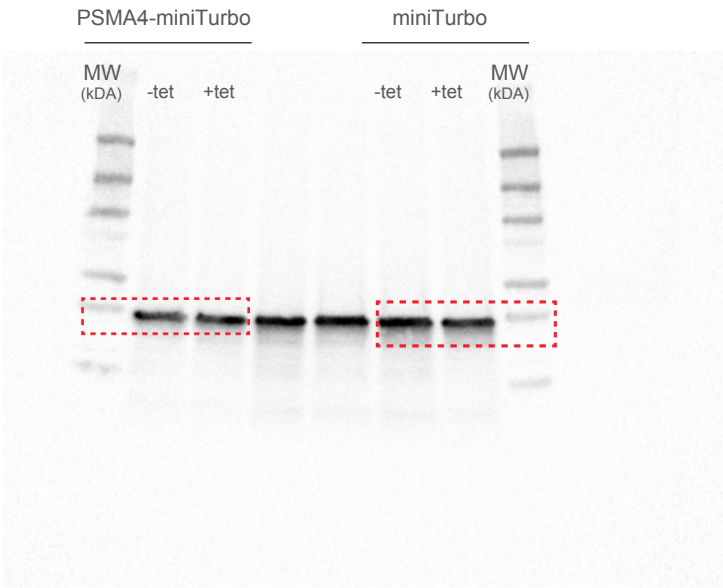

anti-Flag

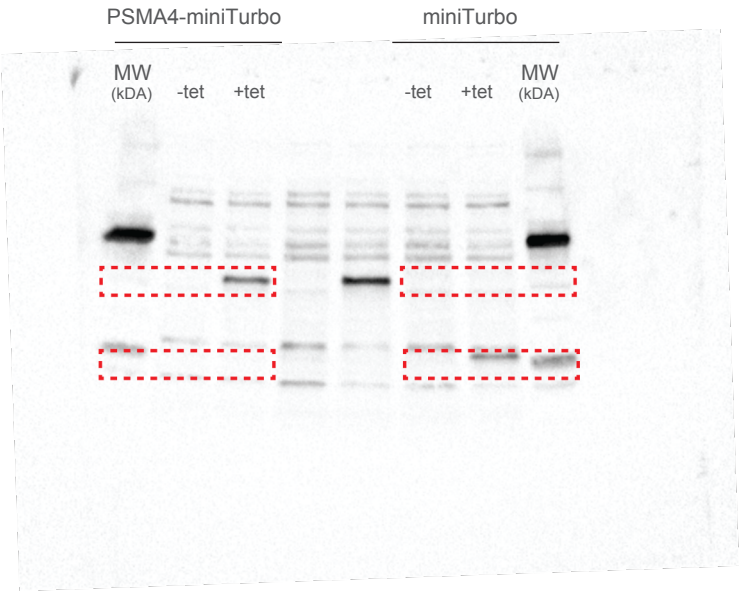

Supplement: Figure 4—figure supplement 1—source data 2. [file elife-93256-fig4-figsupp1-data2.zip › Figure4_SourceData_2/Figure4_S1a.pdf]

Figure 4 figure supplement 1c

ponceau S

streptavidin-HRP

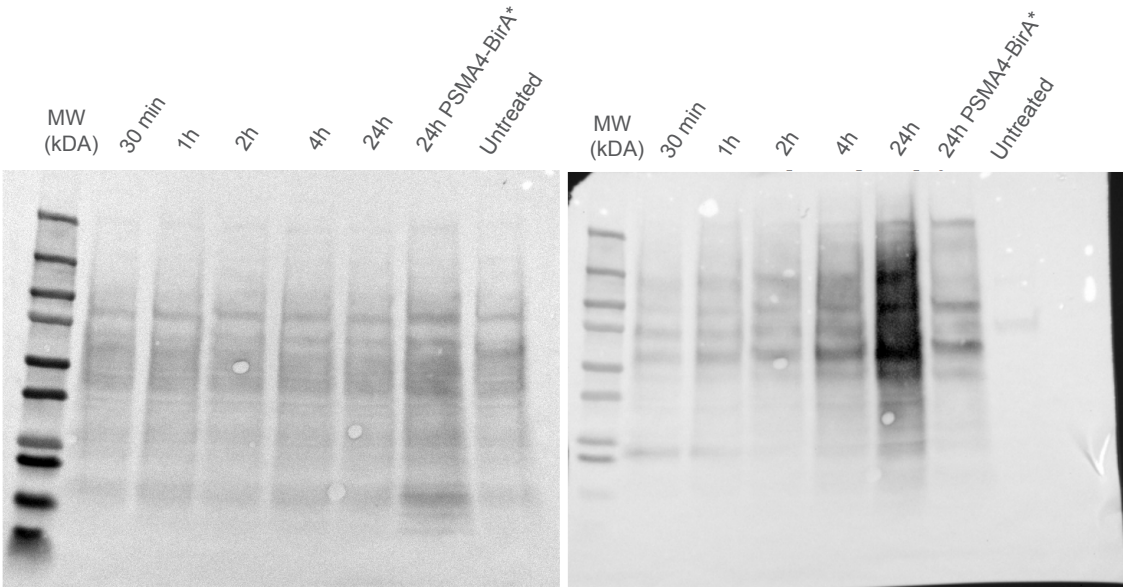

Supplement: Figure 4—figure supplement 1—source data 2. [file elife-93256-fig4-figsupp1-data2.zip › Figure4_SourceData_2/Figure4_S1c.pdf]

Figure 4 figure supplement 1b

anti-GAPDH

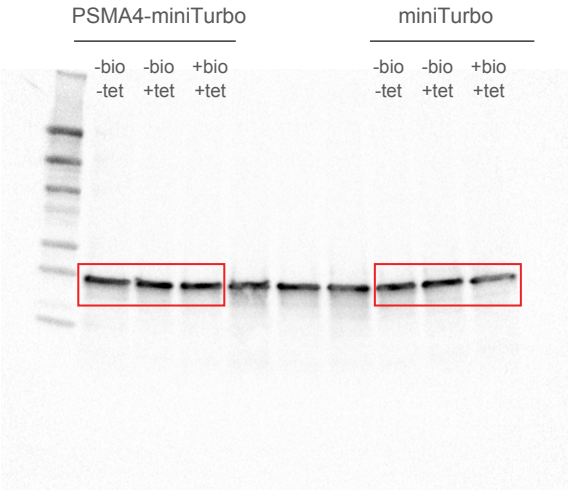

streptavidin-HRP

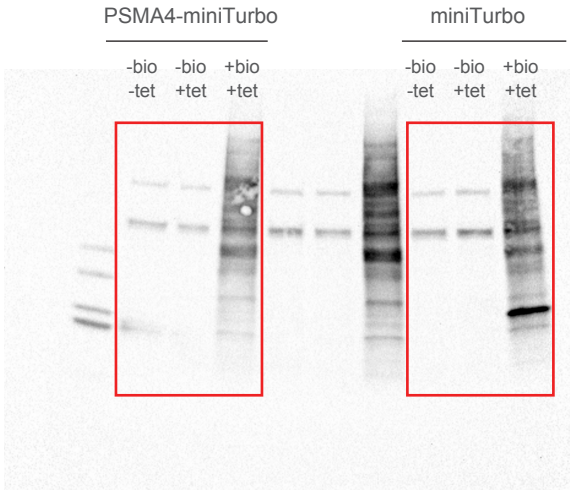

Supplement: Figure 4—figure supplement 1—source data 2. [file elife-93256-fig4-figsupp1-data2.zip › Figure4_SourceData_2/Figure4_S1b.pdf]

**Figure S4j**

anti-MYC

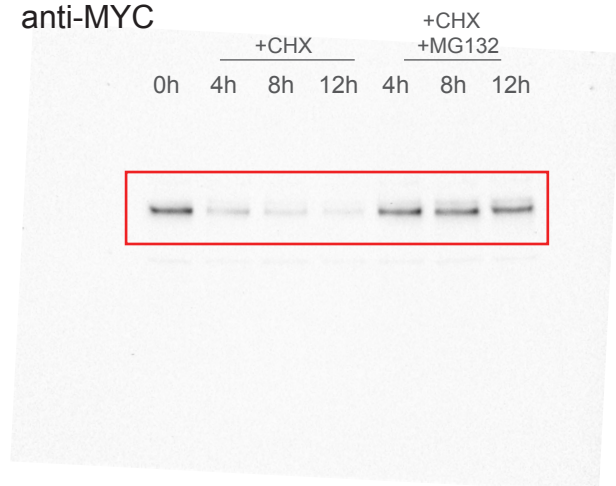

anti-ARMC6

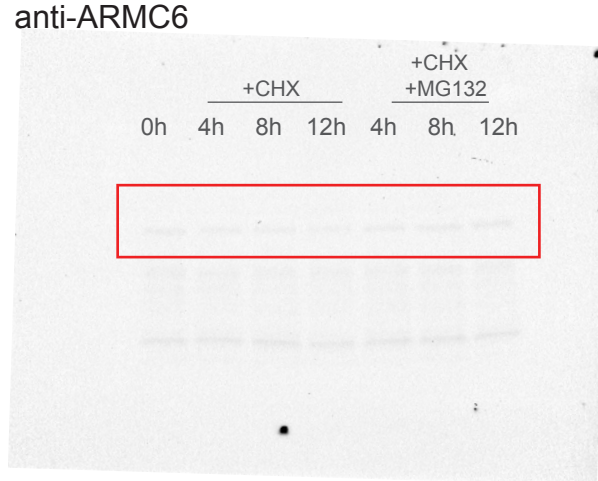

anti-BRAT1

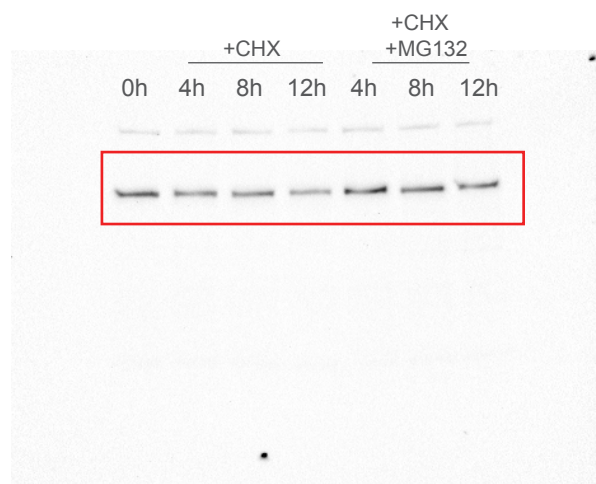

anti-GAPDH

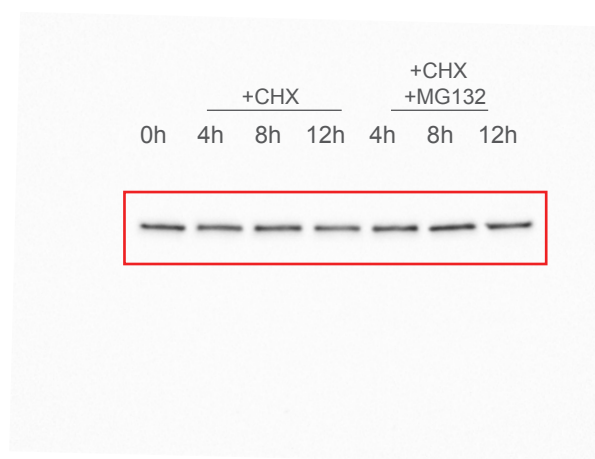

anti-GAPDH (for TIGD5 blot)

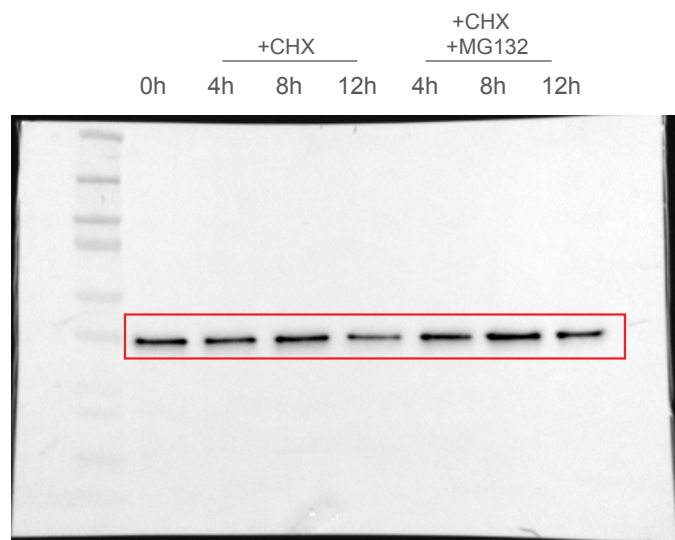

anti-TIGD5

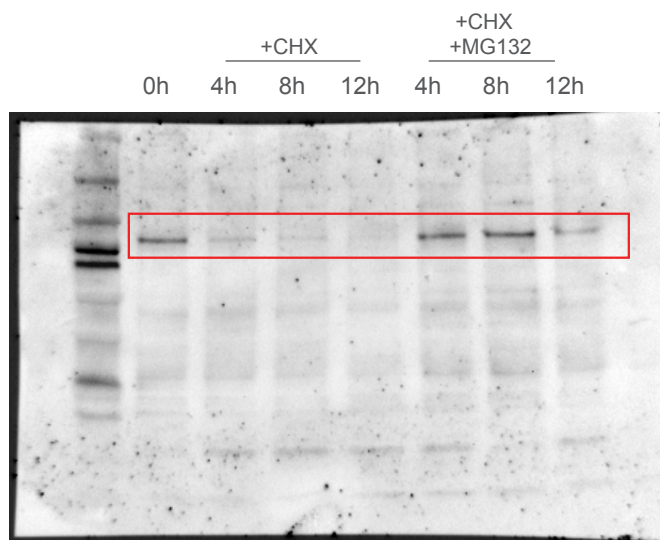

Supplement: Figure 4—figure supplement 1—source data 2. [file elife-93256-fig4-figsupp1-data2.zip › Figure4_SourceData_2/Figure4_S1j.pdf]

Figure 6e

anti-GFP

anti-PSMA4

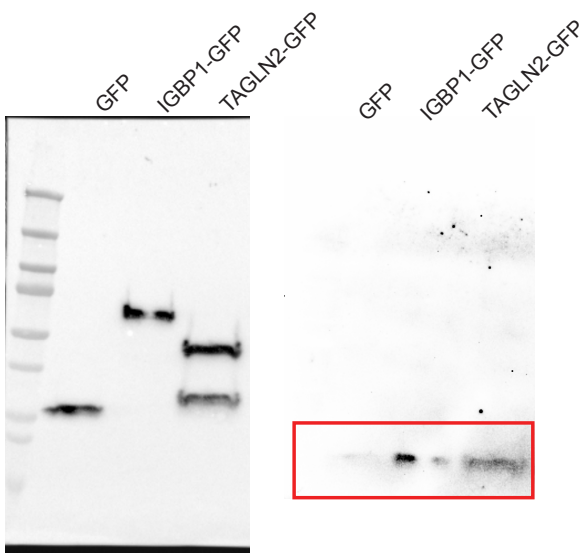

Supplement: Figure 6—source data 2. [file elife-93256-fig6-data2.zip › Figure6_SourceData_2/Figure_6e.pdf]

**Figure 6** anti-PSMA4

**supplemental figure 1a**

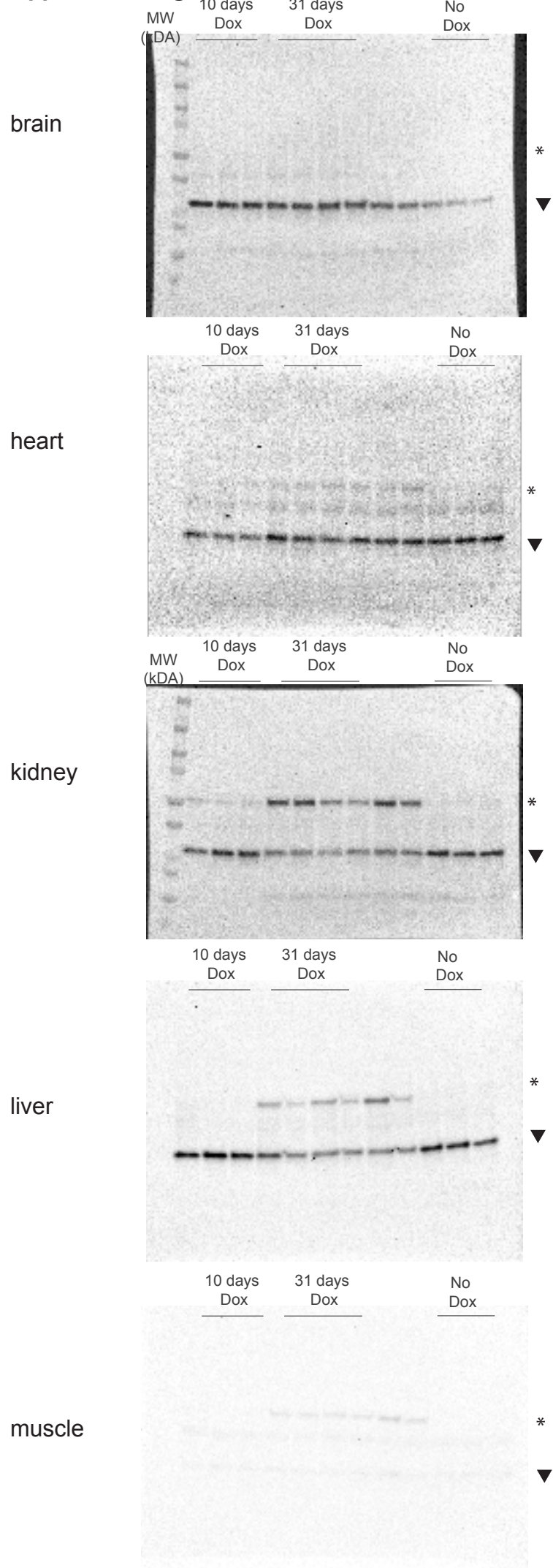

**ponceau S**

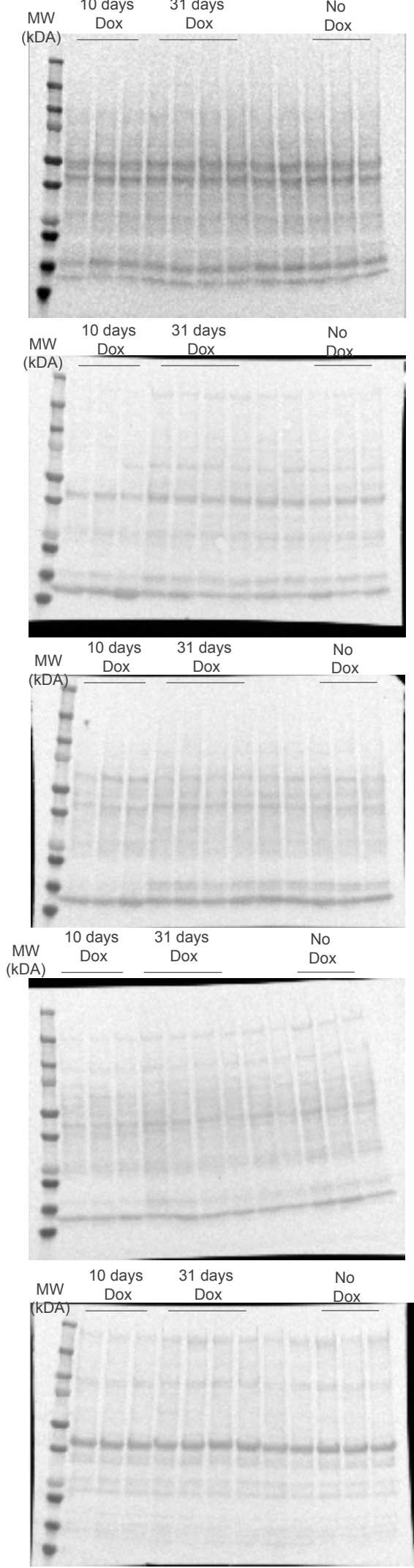

Supplement: Figure 6—figure supplement 1—source data 2. [file elife-93256-fig6-figsupp1-data2.zip › Figure6-figure_supplement1_SourceData_2/Figure6_S1a_.pdf]
