# Supplementary material for: Quantitative mapping of proteasome interactomes and substrates using ProteasomeID
Source: eLife. 2024 Sep 4;13:RP93256. doi: 10.7554/eLife.93256 (PMC11374303; doi:10.7554/eLife.93256)
Supplement: Figure 1—source data 2. [file elife-93256-fig1-data2.zip › Figure1_SourceData_2/Figure_1b_highlighted.pdf]

Figure 1b

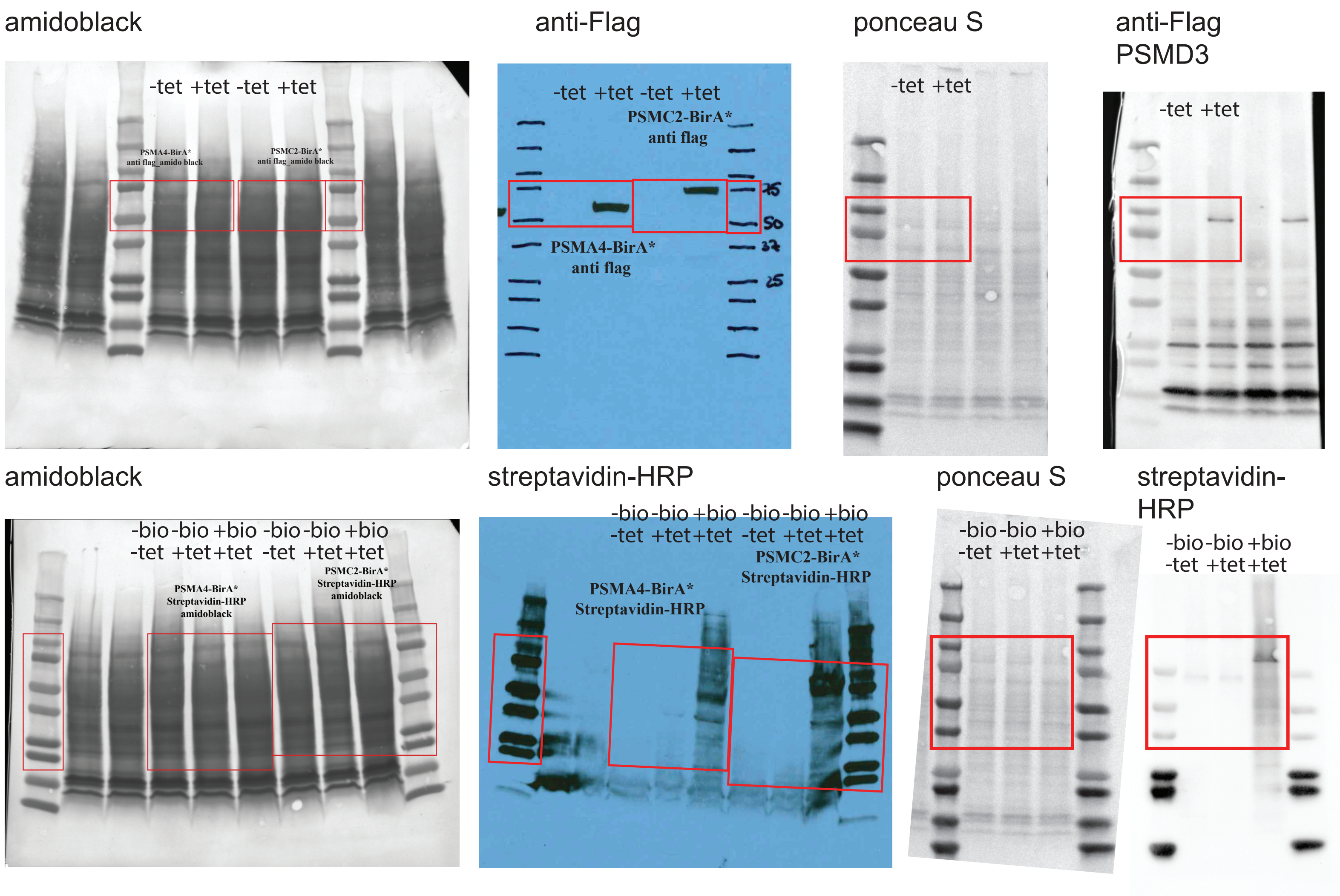

amidoblack
-tet +tet -tet +tet
PSMA4-BirA* anti flag_amido black
PSMC2-BirA* anti flag_amido black
anti-Flag
-tet +tet -tet +tet
PSMC2-BirA* anti flag
PSMA4-BirA* anti flag
75
50
37
25
ponceau S
-tet +tet
anti-Flag PSMD3
-tet +tet
amidoblack
-bio -bio +bio -bio -bio +bio
-tet +tet +tet -tet +tet +tet
PSMA4-BirA* Streptavidin-HRP amidoblack
PSMC2-BirA* Streptavidin-HRP amidoblack
streptavidin-HRP
-bio -bio +bio -bio -bio +bio
-tet +tet +tet -tet +tet +tet
PSMA4-BirA* Streptavidin-HRP
PSMC2-BirA* Streptavidin-HRP
ponceau S
-bio -bio +bio
-tet +tet +tet
streptavidin-HRP
-bio -bio +bio
-tet +tet +tet
