# Supplementary material for: Quantitative mapping of proteasome interactomes and substrates using ProteasomeID
Source: eLife. 2024 Sep 4;13:RP93256. doi: 10.7554/eLife.93256 (PMC11374303; doi:10.7554/eLife.93256)
Supplement: Figure 2—source data 2. [file elife-93256-fig2-data2.zip › Figure2_SourceData_2/Figure_2c.pdf]

Figure 2c

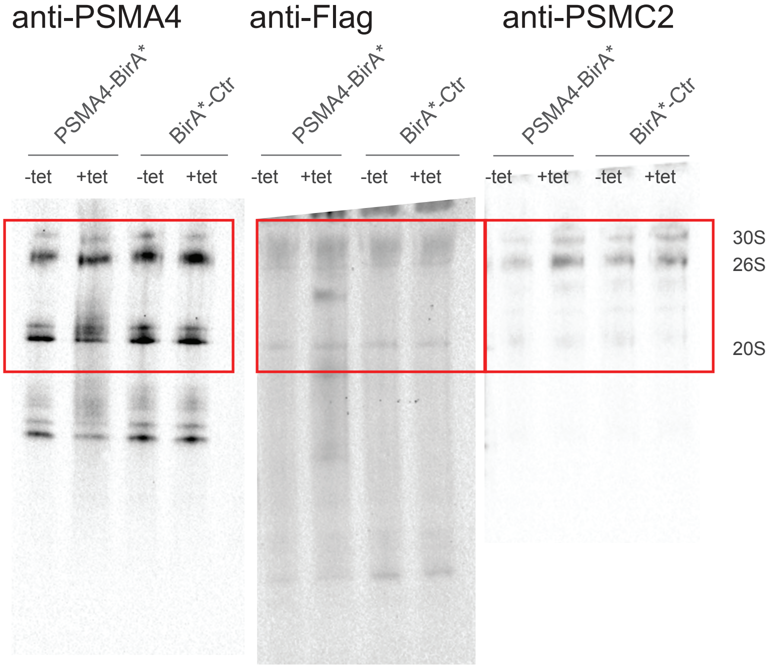
anti-PSMA4
anti-Flag
anti-PSMC2
PSMA4-BirA*
BirA*-Ctr
PSMA4-BirA*
BirA*-Ctr
PSMA4-BirA*
BirA*-Ctr
-tet +tet -tet +tet
-tet +tet -tet +tet
-tet +tet -tet +tet
30S
26S
20S
